# Space and place for health and care – Nationalist discourses in Swedish daily press during the first year of COVID-19

**DOI:** 10.1016/j.heliyon.2024.e27858

**Published:** 2024-03-20

**Authors:** K. von Brömssen, Å. Roxberg, C. Werkander Harstäde

**Affiliations:** a Department of Social and Behavioural Studies, University West, Trollhättan, Sweden; b Department of Health Sciences, University West, Trollhättan, Sweden; c VID, Specialized University, Bergen, Norway; d UiT, University of Tromsø, Campus Harstad, Norway; e Department of Health and Caring Sciences, Linnaeus University, Kalmar, Växjö, Sweden

**Keywords:** COVID-19, Critical discourse analysis, Health, Nationalism, Place, Space, Sweden

## Abstract

Sweden’s strategy during COVID-19 with restrictions but no firm closure of the society surprised the rest of the world and was questioned, not least by neighbouring countries. This article analyses public discourses on space and place for health and care in the Swedish daily press during the first year of the pandemic, 2020. Critical discourse analysis was conducted on daily press newspaper articles to approach issues of space, place, health and care during the COVID-10 pandemic. The findings suggest three main discourses. First, a powerful discourse on unity against the threat is articulated, urging citizens in Sweden to be loyal in the national space. Secondly, an affirming national reconstructing discourse is manifested, related to constructions of borders of national space but also in relation to places of family life and social contacts to ‘flatten the curve’ and stay healthy. Thirdly, later in the period the overarching discourse of the nation and its loyal citizens was torn apart and increasing tensions were articulated due to, as it appeared, the uncertain actions from the government. This study adds to the literature on a theoretical and practical level. Raising awareness on nationalist discourses in relation to place, space, health, and care could prove important in combating inequalities in the local society as well as when cooperating on an international level.

## Highlights

• Use of CDA to analyse newspaper articles on place and health during COVID-19.
• The activation of ‘banal’ and ‘everyday’ nationalism.
• Challenges to the Swedish self-image as modern, unique, and superior.
• Sweden became a battle of surviving the threat, between the known and the unknown.
• Health and care during COVID-19 was above all a matter for the Swedish nation and its citizens.

## 1 Introduction

This article explores newspaper articles in the Swedish daily press to critically analyse discourses on space and place for health and care during the COVID-19 pandemic. This is done by using the framework on space and place drawn from Massey [1,2], supplemented with theories on ‘banal’ and ‘everyday’ nationalism [3,4]. We demonstrate how the nation, or the ‘nationalist imaginary’ [5] (p. 22) became strongly reactivated in Sweden during the COVID-19 pandemic, which prevented people from forgetting the bounded borders of the nation. [cf. 3]. Although nations and nationalism constitute a vast field of research, we argue that there is a continued need to critically analyse the construction of the nation from varied contexts, not least in the dramatic moments of the COVID-19 and how the pandemic affected space and place for health and care. In this way, the present article contributes to the expanding field of research on nationalism, as well as on health and care in post-times of the COVID-19 pandemic.^1^

Accordingly, we argue that nationalism in Sweden was distributed and strengthened through a strong media discourse in a repetitive manner throughout the investigated period.

## 2 Background

Since the beginning of 2020, the world experienced a pandemic caused by severe acute respiratory syndrome coronavirus 2, which came to be known as Corona Virus Disease 2019, or COVID-19 [6]. As of December 2023, almost every country and territory in the world has been affected by COVID-19 and over 6.9 million people have died after contracting the respiratory virus [6].

Sweden’s initial COVID-19 strategy aimed to limit the pandemic, to protect risk categories such as elderly people and to avoid a collapse of the Swedish health care system. Physical distancing was strongly recommended, and mandatory in some situations, but less strict than in many other countries. One example is facemasks that were not mandatory outside health care. The government also made the controversial decision to keep preschools, elementary schools, and lower secondary schools as well as industries open to avoid a total lockdown of society. Therefore, many adults worked more or less as normal [[7], [8], [9], [10], [11]]. The Swedish strategy surprised the rest of the world and was questioned, not least by the neighbouring countries. Denmark presented a picture of an irresponsible Swedish government that was putting their inhabitants' health at risk by keeping schools, restaurants, and sporting facilities open. The decision made by the Public Health Agency of Sweden (PHAS) was to protect, but at the same time maintain, people’s responsibility to keep their distance and remain isolated from others [12]. When comparing the Nordic countries after the first year of COVID-19, it was shown that Sweden had a higher infection rate. Sweden had around 11,000 confirmed infections per 100,000 inhabitants and Finland in comparison had 2500. Sweden also showed a far higher death rate than the other Nordic countries; 140 deaths per 100,000 inhabitants, compared to Denmark’s 45 and Iceland’s 9. However, comparing the number of deaths caused by COVID-19 between different nations can be challenging since how deaths are registered can vary from country to country [13].

A structural explanation of why the Swedish corona strategy stood out, from an international perspective, can be found in Swedish authorities’ autonomous position towards government departments, which is stated in the constitution of Sweden [7,[14], [15], [16]]. The head of the social department cannot decide over the decisions that PHAS makes. PHAS, on the other hand, is obliged to follow the laws and rules set by the government but can apply them independently. Additional contingent factors like weak political leadership and strong bureaucratic leadership in recent years have been mentioned as factors behind the Swedish strategy [7].

Accordingly, it is often argued that Swedish citizens for long have had a great amount of trust, both towards each other and towards the Swedish state [[17], [18], [19]]. When studying previous health crises, such as H1N1 (the “swine flu”), it is clear how the government relied on Swedish citizens' trust in the state and how the citizens showed solidarity with the decisions that were made [20]. , Giritli Nygren and Olofsson [20], showed that the Swedish COVID-19 strategy became part of what could be seen as an institutionalized risk management that contributed to a sense of national identity building. When this faith, responsibility, and solidarity that forms the Swedish identity was observed from the outside, this threatened the trustworthiness of Sweden [20]. Giritli Nygren and Olofsson [20] used the concept of exceptionalism as a descriptor of the Swedish citizens’ high level of trust in institutions and politicians, something that can link the Swedish strategy together with a common belief and self-image of Sweden as modern, unique, and superior to other countries [cf. 21,22]. As shown, Sweden handled the pandemic in a partly different way compared to large parts of the rest of the world. It is therefore interesting to investigate how the Swedish media (newspapers) portrayed the first year with COVID-19.

Given this background, the aim of this article was to critically analyse discourses on how place and space for health and care was discursively constructed in the two main Swedish national newspapers during the early stages of COVID-19 pandemic in Sweden in 2020.

The remainder of this paper is organized as follows. In the first section, we present previous research followed by the theoretical framework that informed our work, namely the concepts of space and place, health and care, complemented with theories on the nation, which, during analysis, came through as a productive conceptual lens for explaining our data. We then introduce the methodology, followed by a section of our findings under three headings, namely ‘United against the threat – loyalty to the nation’, ‘Reconstructing national borders and the Swedish strategy debate’, and ‘Increasing tensions in the national discourse’. Finally, we discuss our results.

## 3 Previous research

Previous research on Sweden during the COVID-19 pandemic has sought to explain various aspects of the pandemic and the Swedish strategy from the specific Swedish governance model and the Swedish authorities’ autonomous position with small ministries, relying on experts rather than politicians, as discussed above. The idea is that these bureaucratic agencies make independent assessments based on the best available knowledge and proven experience and that the government listens to the bureaucratic experts [2,7,8,11,14]. Thus, research on governance discusses how the relationship between the government and the bureaucracy in Sweden is different to that in other countries. These differences became very clear during the pandemic and played a significant role in the Swedish strategy. In much of this research, political trust in public institutions, in the pandemic policies, and in health authorities are used as explanatory models [23,24].

Other aspects of the COVID-19 pandemic are covered by analysis of the geography of COVID-19 in Sweden and the question of why COVID-19 hit some places harder than others; this is an interesting question in relation to analysis of space, place, and health [25]. Florida and Mellander discussed this issue and stated that factors associated with diffusion mattered more than place-based factors. Thus, COVID-19 was more prevalent in places that were hit early, and in places proxy with higher levels of infections, which is related to diffusion of the virus. The most significant place-based factor was the presence of high-risk nursing homes, which outperformed all other socioeconomic variables in Florida and Mellander's [25] research.

Analysis of the media debate is especially interesting in relation to the focus of this article, but also as much previous research has pointed to, the power of media in representations [cf. 26]. Newspapers provide a powerful discursive space for constructions of identities, such as national identities [3]. Andersson and Aylott [7] stated that there was a ‘burst of nationalism in parts of the media’ (p. 1) during the pandemic, as well as the theme of ‘Swedish exceptionalism’ [11,22,27,28]. In the field of media Hagström et al. [29], explored various narratives and ‘Othering of Sweden’ in American, German and Nordic media reporting on Sweden in the first eight months of the COVID-19 crisis. They showed how the narratives on Sweden and COVID-19 drew on previously established narratives resulting in four different logics of othering: emotional, strategic, analytic, and nuanced. Also Martikainen and Sakki [29], explored how Sweden was depicted abroad, identifying how COVID-19 was recontextualised in Finnish newspapers and what types of stereotypic conceptions were drawn upon intertextually. Martikainen and Sakki [29]identified three multimodal rhetorical strategies: namely, those related to moralising, demonising, and nationalising Sweden. These strategies constructed discourses of an arrogant, immoral, and dangerous Sweden and generated distinct group boundaries between Finns and Swedes. These constructions reflect a historical and traditional stereotypical conception of Finland and Sweden as rivals [29]. It was investigated how Sweden attempted to frame its COVID strategy in a positive light in international media [[30], [31]], where Sweden evolved rather rapidly ‘from bold to pariah’ [31 p. 54] and was depicted as an outlier with discriminating values and practices. To the research on media and nationalism in Sweden we add work by Gustavsson and Larsson Taghizadeh and Idevall and Bellander [31,32]. The former asked why, in the polls, a majority continued to support Sweden’s internationally deviant model of the pandemic. Their analysis showed that this was a result of a ‘rally around the flag’ (RATF), where ‘Swedishness’ was at stake. The researchers argued that institutional national pride, uncritical patriotism and national chauvinism predicted support for the Swedish strategy [31]. It was also concluded, following analysis within a theoretical framework of populism and nationalism, that both phenomena have ‘flourished in the case of COVID-19’ [33 p. 105]. Idevall Hagren and Bellander [32]. analysed newspaper articles and showed how a nationalistic framing highlights trust and responsibility as key aspects of the strategy, but also how trust and responsibility are used in delegitimations such as consequences for individuals' everyday lives. Finally, it is worth discussing the concept of ‘pandemic nationalism’ that Triandafyllidou suggested [34]. This concept is seen as a response to the increased challenges from globalisation by incorporating some in the nation, ‘those who serve the community’, and excluding and othering ‘those who threaten wellbeing’. This might reflect ‘a turning point that signals a new phase of development of nationalism’ [34]. In sum, this research overview has shown that there is an increased interest in research on COVID-19 and nationalism, not least on critical studies to which this article adds. However, as this research overview has shown, space, place and health as conceptual tools are largely missing in these analyses. This is something that we wish to highlight in our analyses.

## 4 Theoretical framework

Space and place are concepts that are widely used in many disciplines as well as in health and caring sciences. In contemporary theorizing, space and place are often understood in relational and topological terms, as meaning something actively involved in human, as well as non-human becomings [35]. In line with this, Massey’s work has contributed to a significant rethinking of space and place as the product of relations rather than as fixed and stable arrangements. Space is constructed through dimensions of multiplicity, always unfinished and under construction, as argued by Massey. There is also the idea that space and politics are co-constitutive and operate at multiple and complex levels [1,2]. These ideas are quite different from earlier research, where both space and place denoted a more passive backdrop for human social life and functioned as passive containers, or frames of reference, from which matters of health and care transpired [36]. Thus, how we analyse and use the concepts of space and place is important for how we understand care and health [35].

To analyse our data, we also turned to Billig’s work on ‘banal nationalism’ [3]. This well-known and very much used concept describes the everyday, less visible forms of nationalism, which become absorbed into the nation’s environment and are neither grand, exotic nor distant. ‘Banal nationalism’ emphasizes the nation as a social condition, achieved through constant daily repetitions of names, borders, languages, during sport events, in national songs and through many other popular expressions. Billig used the term ‘national space’ for this construction [3,37] and argued *inter alia* that it is often the ‘small words’ that remind us that we live in a nation. Billig emphasized a language perspective and argued:

Small words rather than grand and important memorable phrases make our national identity unforgettable. To explore such matters, we should not only pay attention to words like ‘people’ or ‘society’ but also become linguistically microscopic’, since the secret of banal nationalism lies in tiny words such as ‘we’, ‘this’ and ‘here’. (Billig, 1995, pp. 93–94)

In the present article, the concept of ‘banal nationalism’ is supplemented with the concept of ‘everyday nationalism’ [4]. The everyday nationalism approach builds on Billig’s work [3] on banal nationalism but differs in its focus on understanding the meaning and experiences of nationhood from the perspective of human agency, such as people on the ground. ‘Banal nationalism’, by contrast, explores the state-centric top-down conception of nationhood [4, 37].

An interest in language [3] was also underlined by Fairclough [26,[38], [39], [40]] in his work on critical discourse analysis (CDA), which we use in our analysis and turn to now.

## 5 Methodology

Critical discourse analysis (CDA), at a level of detailed textual and thematic analysis with an inductive approach, was chosen for this study. CDA is a transdisciplinary approach used to analyse the role of language in the construction of knowledge, ideology, and power [39] which was appropriate for this study. CDA takes several different approaches and a variety of methods that depend on research goals and theoretical perspectives. Like most discourse analyses, CDA regards discourse as being socially constructed and contextually contingent. As Fairclough pointed out, discourses are multilayered, relational, and constructed in communication between people who talk, write and communicate with each other, but also describe relations between concrete communicative events such as newspaper articles [26]. In this article, we draw on the three-dimensional model from Fairclough [26] and Chuliaraki and Fairclough [41] – namely description, interpretation, and explanation – to analyse media discourse concerning COVID 19. The first dimension in this framework, *description*, focuses on important features of the text such as grammar, structure of the agent, passive patterns sentence, etc. It is about the grammatical and semantic choices of an author that can be directly observed in the data [42]. The second stage, *interpretation*, considers the more general ways of using language that underlie a text, such as processes of text production, distribution and consumption, where quotations from various sources are important. Lastly, *explanation* involves scrutinising the broader social context in which textual and discursive dimensions reside, where we ask about the wider social effects of the linguistic structures; thus, what is told about COVID 19 in Sweden in the beginning of the pandemic in 2020 – and, importantly, what is included and not included in the text. As Fairclough argued, a discourse is a complex of these three elements and there is ‘a significant connection between features of texts, ways in which texts are put together and interpreted, and the nature of the social practice’ [26 p. 74, 43 p. 87]. Thus, CDA researchers are recommended to make links to structures, institutions, and dominant ideologies in society that are imbued in discursive constructions, such as politics and economy [44].

Accordingly, a CDA approach is beneficial for uncovering the apparent and less apparent articulations that take place in language in social processes by exploring the connections between specific discourses and the wider social context [26,[38], [39], [40]]. In the present article, this is done by focusing on the discursive constructions of space and place for health and care in daily press during the initial stage of the pandemic COVID-19 in Sweden. Thus, our analysis is concerned with the construction of public discourses and ‘ways of talking’ and ‘ways of seeing’ [39, p. 43]. Its primary focus is not on single entities or individuals but on social relations and communication constructing public discourses [39]. To achieve a high level of transparency and credibility, the research group worked together in all parts of the analysis to ensure that each discursive theme was explored thoroughly and critically. This cooperation also made it possible to jointly reflect and validate the process.

The corpus for the study was based on analyses of articles from the two main Swedish morning newspapers, *Dagens Nyheter* (*DN/‘Daily News’*) and *Svenska Dagbladet* (*SvD/‘The Swedish Daily Newspaper’*; our translations). The media model in Sweden can be characterized as a ‘neoliberal media welfare state’ [45], although this is currently under pressure due to digitisation and international competition. *DN* and *SvD* are quality-oriented morning newspapers and dominate the market [46]. *DN* is an ‘independently liberal’ newspaper that aims to be the opinion-leading newspaper in Sweden. *SvD* describes itself as independent of political, religious, commercial, and individual interests and aims to initiate and constitute an open arena for free debate in a humanist way. These two newspapers reach 1.9 million readers daily and have approximately 1.5 million unique online readers each day [47].

The newspaper articles for the present study were collected through an online search in a national digital archive called Mediearkivet (Retriever), the largest digital archive of media sources in Scandinavia, where it is possible to conduct online full-text searches. We performed a search for articles from the 15th of each month from March until December 2020 focusing on the aim of this study. The initial year was chosen because the crisis, both personal and global, was a new and challenging experience for most of the inhabitants in Sweden and reveals the introductory reactions to the covid threat. The choice to capture the media coverage on the 15th of each month was made to get a picture of the situation several times during the first year of COVID-19 and to investigate how the textual descriptions developed. The keywords used in the searches were (Space OR Place OR Health OR Ill* OR Environment) AND (Corona OR COVID-19 OR Pandemi* OR Virus). The data material consisted of 65 news reports, as summarized in Table 1. These reports were chosen from the perspective that they responded to the purpose of the study. Most of the authors were journalists, but several articles were written by ministers and party leaders, as well as scientists from different disciplines. The data material was seen as a whole, where various texts were assembled as one single document. Therefore, statements in individual texts have lesser implications and are instead seen as exponents of a discourse formed in the sociocultural practice, in accordance with Fairclough’s theoretical assumptions [38,39]. The discourse examples in the present article should be viewed as representative of the media discourse that is consistently at work in society [cf. 48, 49].

**Table 1:** List of documents used in the discourse analysis.

Author	Title	Date	Source
Almgren, J.	Privata vårdjättar slår tillbaka mot kritiken [*Private healthcare giants are fighting back against the criticism*]	15 May 2020	**SvD**
Andersson, M.	Vårt reformarbete fortsätter vid sidan av krisåtgärderna [*Our reform work continues alongside the crisis measures*]	15 April 2020	**DN**
Arvidsson, J.	Pandemin kom i vägen för Filippas och Laurisas planer för framtiden [*The pandemic got in the way of Filippa and Laurisa’s plans for the future*]	15 November 2020	**SvD**
Björkman A. Q.	Coronakris och flyktingkris - en giftig kombination [*Corona crisis and refugee crisis – a toxic combination*]	15 March 2020	**SVD**
Brunnberg K.	Isolerad? 5 kulturtips för att stå ut [*Isolated? 5 cultural tips to endure*]	15 March 2020	**SVD**
Brännström, S.	“Vi är inte rustade alls – kan bli smittade allihop” [*‘We are not equipped at all - we can all get infected’*]	15 April 2020	**SvD**
Brännström, S.	Facken: Arbetsgivarna tar inte coronaoron på allvar [*The Unions: Employers are not taking the corona virus seriously*]	15 April 2020	**SvD**
Carp O.	Statens sms väcker kritik - därför saknades länken [*The text message from the State arouses criticism – therefore the weblink was missing*]	15 December 2020	**DN**
Editorial	Intensivvård på hög nivå [*Intensive care at a high level*]	15 April 2020	**SvD**
Editorial	Klarspråk krävs om den svenska strategin [Plain language is required about the Swedish strategy]	15 May 2020	**DN**
Editorial	Många personer på kirurgavdelning smittade [*Many people in the surgical ward were infected*]	15 April 2020	**DN**
Editorial	Sverige har passerat 1000 dödsfall i COVID-19 [*Sweden has passed 1000 deaths from COVID-19*]	15 April 2020	**SvD**
Editorial	Sverige skulle skydda de äldre men svek dem istället. Coronakrisen [*Sweden was supposed to protect the elderly but failed them instead. The Corona crisis*]	15 October 2020	**DN**
Editorial	Vart tog vänsterns klassperspektiv vägen? [*Where did the left wing’s class perspective go?*]	15 November 2020	**DN**
Dragic, M.	Familjen fick en vecka på sig att tömma bostaden efter faderns … [*The family was given a week to empty the home after the father’s …*]	15 May 2020	**DN**
Eriksson, H.	Överläkaren om intensivvårdens pressade läge: “Ett otroligt tryck” [*The chief physician on the pressured situation in intensive care: ‘Unbelievable pressure’*]	15 April 2020	**SvD**
Eriksson H.	Ovisst för strandsatta svenskar i USA: “Är en helt ny situation” [*Uncertain for stranded Swedes in USA: “A completly new situation”*]	15 December 2020	**SVD**
Ewald, H. et al.	Strategier oförändrade trots färre smittade [*Strategies unchanged despite fewer infected*)	15 May 2020	**DN**
Fakta/Perspektiv 2020	Lågt förtroende för FHM i utsatta områden: “Svårt få ut information” [*Low trust in PHAS in vulnerable areas: ‘Difficult to get information out’*]	15 June 2020	**SvD**
Falkirk J.	Svenskarna har stort förtroende för Folkhälsomyndighetens agerande [*The Swedes have great confidence in the Public Health Authority*]	15 March 2020	**SVD**
Frejdeman, H.	Inreseförbudet till Sverige förlängs [*The entry ban to Sweden is extended*]	15 May 2020	**SvD**
Gustafsson, A.	Äldreboenden granskas efter smittspridning [*Nursing homes are inspected after spread of infection*]	15 April 2020	**DN**
Gustafsson, A. & Röstlund, L.	Region Stockholm tillsätter dubbla utredningar om vård av äldre [*Region Stockholm appoints double investigations into care for the elderly*]	15 September 2020	**DN**
Gustafsson, H.	När Förbudssverige blev det oansvariga landet [*When Prohibition Sweden became the irresponsible country*]	15 May 2020	**SvD**
Hansson A.	Förvirrad stämning efter att Danmark stängt för omvärlden [*Confused mood after Denmark closed to the outside world*]	15 March 2020	**DN**
Hansson, S.	“Inför striktare regler i tre till fyra veckor” [*‘Introduce stricter rules for three to four weeks'*]	15 April 2020	**DN**
Holmgren, M.	“Inget misslyckande att andra länder stänger gränsen mot oss” (Statsministern) [*‘No failure that other countries close the border against us' (Prime Minister)*]	15 June 2020	**DN**
Holmgren, M. & Olsson, G.	Forskare om skepsisen: En jättebra början om 70 procent får vaccinet efter nyår [*Researchers on the skepticism: A great start if 70 percent get the vaccine after the New Year*]	15 October 2020	**DN**
Jensen, C.	Vi behöver ett nytt samhällskontrakt [*We need a new social contract*]	15 April 2020	**DN**
Johansson, K. et al.	Sverige saknar etiskt ramverk för beslut vid pandemier [*‘Sweden lacks an ethical framework for decisions in pandemics'*]	15 May 2020	**DN**
Karén F.	Trovärdighetens dag för coronakommissionen [*Credibility day for the corona commission*]	15 December 2020	**SVD**
Karén F.	Brynäs tränare missar omstart: “Rejält sjuk” [*Brynäs’ coach miss restart: “Seriously ill*”]	15 December 2020	**SVD**
Karén F.	Nio av tio tänker följa Löfvens julråd enligt SvD/Sifo-mätning [*Nine out of ten intend to follow Löfven’s Christmas advice according to SVD/Sifo-measurement*]	15 December 2020	**SVD**
Karén F.	Tryckkammare stängs – trots forskningen [*Pressure chamber is closed – despite the research*]	15 December 2020	**SVD**
Karén F.	De är vinnare och förlorare när den stängda gränsen stoppar kunderna [*They are winners and losers when the closed border impedes the customers*]	15 December 2020	**SVD**
Karén F.	Stockholms intensivvård överbelagd – regionen går upp i förstärkningsläge [*The intensive care in Stockholm is overcrowded – the region goes up in gain mode]*	15 December 2020	**SVD**
Karén F.	Därför är coronavaccinet inget att oroa sig över. [*Therefore, the corona vaccine is nothing to worry about]*]	15 December 2020	**SVD**
Kudo, P.	Räkna med hemmajobb I ost – experterna om coronaläget [*Expect home work this autumn – the experts on the corona situation*]	15 Aug 2020	**SvD**
Kudo, P.	Det får vi göra i sommar – experternas tre scenarier [*This we can do this summer – the experts' three scenarios*]	15 May 2020	**SvD**
Lindholm, A.	Alla stockholmare ska få möjlighet att göra coronatester [*Everyone from Stockholm shall have the opportunity to take corona tests*]	15 June 2020	**DN**
Lindkvist, H. & Ahlström, K.	Så kommer midsommar att splittra svenskarna [*So, Midsummer will divide the Swedes*]	15 June 2020	**DN**
Lianke Y.	När föreställningen är över måste vi vårda minnet av coronas offer *[When the performance is over, we must cherish the memory of the corona’s victims*]	15 March 2020	**SVD**
Lindblad A.	Hur ska idrotten kompenseras? *[How should sports be compensated?]*	15 March 2020	**SVD**
Ludvigsson, J.	Svensk öppenhet räddar fler liv på sikt [*‘Swedish openness saves more lives in the long run’*]	15 April 2020	**DN**
Ludvigsson, J.	Vi kan klara pandemin utan lockdown [*We can cope with the pandemic without a lockdown*]	15 April 2020	**SvD**
Löfven, S.	Vår strategi har inte misslyckats [*Our strategy has not failed*]	15June 2020	**SvD**
Majlard, J.	Hemisolering införd: “De flesta är positiva” [*Home isolation introduced: ‘Most are positive’*]	15 October 2020	**SvD**
Manzoor, A.	Det här är flockimmunitet – och därför är det … [*This is herd immunity – and therefore it is …*]	15 May 2020	**DN**
Nerbrand, S.	Coronakrisen: Hatten av för vårdare av äldre [*Corona crisis: Hats off to carers of the elderly*]	15 August 2020	**DN**
Olsson, H.	Hallengren: “Det gäller att vara förberedd på olika scenarion” [*Hallengren: ‘It is important to be prepared for different scenarios'*]	15 November 2020	**DN**
Olsson K.	Venstres partiledare: “Sverige kommer att följa Danmark” [Liberal party leader: “Sweden will follow Denmark”	15 March 2020	**SVD**
Ritzé, J.	Folktomma gator minskade bullret – tystare än på julafton [*Empty streets reduced the noise -quieter than on Christmas Eve*]	15 September 2020	**DN**
Rockström, J.	Fler katastrofer pyr under den svenska idyllens yta [*More disasters smolder beneath the surface of the Swedish idyll*]	15 August 2020	**SvD**
Rosén, H.	Fack och arbetsgivare kräver större krispaket [*Unions and employers demand a larger crisis package*]	15 April 2020	**DN**
Rutberg, H.	Vart tog försiktighetsprincipen vägen? [*Where did the precautionary principle go?*]	15 May 2020	**SvD**
Stenberg, E.	Den svenska krisberedskapen skakar i grunden [*Swedish crisis preparedness is shaking to the core*]	15 March 2020	**DN**
Stenberg E.	Pandemin kommer att ändra krisberedskapens grundbultar [*The pandemic will change the foundations of crisis preparedness*]	15 March 2020	**DN**
Svensson P.	Är vi klokare i dag än på spanska sjukans tid [*Are we wiser* today than in the days of the Spanish flu]	15 March 2020	**DN**
Swedish national news agency	Handplockade specialgrupper ska stoppa smittan inom hemtjänsten [*Hand-picked special groups will stop the infection in home care*]	15 April 2020	**DN**
Swedish national news agency	Färre söker hjärtvård [*Fewer seek cardiac care*]	15 April 2020	**DN**
von Hall, G.	Pandemin en kris på flera plan – våg av psykisk ohälsa kan vänta [*The pandemic a crisis on several levels – wave of mental illness can be expected*]	15 May 2020	**SvD**
Yttergren, I.	Viruset driver utvecklingen framåt [*The virus drives development forward*]	15 April 2020	**DN**
Yttergren, I.	Företagshälsovården glöms bort i virusbekämpningen [*Occupational health care is being forgotten in the fight against viruses*]	15 April 2020	**DN**
Wolodarski P.	Stäng ned Sverige för att skydda Sverige [*Shut down Sweden to protect Sweden*]	15 March 2020	**DN**
Wolodarski P.	Välfärden och coronkrisen i centrum av budgetdebatten [*Welfare and Coroncrisis in the center of the budget debate*]	15 December 2020	**DN**

To make the process in the analysis transparent and distinct, we drew on Svensson’s systematic work on discourse analysis in five steps [50]:

• The empirical data were first scanned through and then explored more thoroughly to detect details and possible contradictions, as a way of becoming acquainted with the material.
• The text was then searched through for parts that corresponded with the aim of the study, as a way of organising the empirical data. Each of these parts was marked with a word (code) that expressed the meaning.
• These parts and codes were then read again carefully to answer the question: ‘What happens here?‘, in what is called ‘close reading’. It was important to keep an openness to the texts to detect details and aspects that might not be expected and to encourage creative thoughts about the data. Some words surfaced as richer and more important than others to the discourse of space and place for health and care. Examples were home, borders, contamination, distance, isolation, and security, as well as text units connected to these words. These text units also showed different stances of power and ideological positionings.
• The data material was then categorized and labelled based on the aim of the study, where similarities, differences and nuances were captured.
• Finally, we took a step back to achieve distance and gain an overview. The analysed text was then put into its social context to understand the role and function of the language and discourses on space and place for health and care in the Swedish society during the initial phase of COVID-19.

## 6 Findings

The analysis of the media texts suggested that there were three overarching and competing discourses of space and place for health and care in Sweden during 2020: (1) United against the threat – loyalty in the national space, (2) Reconstructing national borders and the Swedish strategy debate, and (3) Increasing tensions in the nationalist discourse. We begin this section with an analysis of the most strongly articulated discourse initially in March 2020. To illustrate each dominant discourse, representative extracts of these discourses from newspaper articles were selected. Additionally, we discuss what ‘realities’ that are being (re)produced with the help of the theoretical framework and previous research in the field.

### 6.1 United against the threat – loyalty in the national space

In March of 2020, newspapers described COVID-19 as creating a totally unknown situation. ‘The truth is that no one knows’ (Cederblad, SVD, 15th March 2020), which created fear. Moreover, the Swedish Prime Minister Stefan Löfvén reinforced these articulations, stating about the pandemic ‘It is the greatest threat against public health for many centuries’ (Stenberg, DN, 15th March 2020). The newspaper analysis revealed a discursive overall structure that positioned the pandemic as a huge threat, and how health and care was seen as a matter for all citizens articulated as ‘for the whole of Sweden’. The threat was at the same time fortified by the calls for all the political parties to take a united responsibility to the threat, both within and between countries. This was *inter alia* articulated in the beginning of March 2020:

The whole of Sweden now unites in the struggle against the Corona virus. The pandemic forms a threat against people’s life and health […]. The virus outbreak and its aftermath shall be met by cooperation and joint responsibility, both within and between countries.

(Andersson, DN, 15th March 2020).

As that quotation indicates, the concept of Sweden as an imagined community (Anderson, 2016/1983) and a national space is self-evident when challenged by ‘a threat against life and health’. There is a call for work to be done ‘both within and between countries’, followed by words of agreement and consensus such as ‘cooperation’ and ‘joint responsibility’. Thus, a strong unifying discourse is formulated at the beginning of the pandemic and the bounded borders of the Swedish nation, and its inhabitants are repeated to meet the pandemic threat.

We note how Sweden as a national space at the textual level is reconstructed and addressed as being a bounded and united space in the struggle. Sweden’s minister of finance, Magdalena Andersson, repeatedly articulated this construction of a united Swedish nation by giving prominence to vigorous emergency measures to fight against the effects of the COVID-19 contamination. Andersson articulated that the government had suppressed the serious consequences for businesses, employment and the national economy (Andersson. DN, 15th March 2020), and she used a strong discourse supporting the nation. The threat against the whole and allied nation was met by appeals from ministers and party leaders and reinforced by calls to moral responsibility, now by each of us:

We should all help to ‘flatten the curve’, that is keeping the virus from spreading as much as possible. That could be the most important moral decision each of us has to make. (Rutberg, 15th May 2020).

The appeals are directed to the nation, which is seen as a bounded and fixed space, and to every person’s responsibility, constructed as a ‘we’ of the nation [cf. 3]. This construction is twofold, and as such directed to each Swede and to the nation that each Swede belongs to. This discourse is unifying and, as such, meant to prevent opposition from different actors in various places and to form one space: the nation. Moreover, the construction of a Swede is that of a responsible person included in a responsible nation and the nation turns into a symbiosis of the Swedish inhabitant in the overarching and dominating discourse during the start of the pandemic.

The loyalty to the nation of Sweden was a discursive construction formulated as obligations for inhabitants to follow and to avoid the invisible threat, both for themselves, the family, and the nation. This loyalty was tested as restrictions must be followed, which constructed the national space as a place for health and caring. It also showed personal pride in being part of and united with the nation Sweden and contributed to the national welfare. The texts used words like ‘we’, ‘our’, and ‘all’ to build on that loyalty among all inhabitants to the nation. Such loyalty leads to the inclusion of inhabitants, but also to the exclusion of non-inhabitants or non-Swedes through the emphasis on strengthening borders against other nations that were reinforcing the notion of the self-evident national space.

### 6.2 Reconstructing national borders and the Swedish strategy debate

A second overarching discourse in the analysed newspapers during the COVID-19 pandemic consisted of articulations related to different forms of borders. This discourse is extensive in the texts, as Sweden’s borders were reinforced during the pandemic, to other nations, to borders within the nation, and personal borders related to family life. Thus, borders were materialized in relation to national space but also in relation to places of family life and social contacts to ‘flatten the curve’ and stay healthy [51]. The government and PHAS set rules for how to travel, how to see each other, who to meet and how to work and live (Kudo, SvD, 15th May 2020). At the same time, these rules in Sweden were usually not mandatory, but instead articulated as ‘recommendations’, due to the Swedish constitution as discussed above, which prohibited a shutdown without legal support. This constitutional rule was often explained by the fact that Sweden had not been at war or serious crisis for a very long time, and therefore did not have any such exceptional legislation (SVT News, 2nd April 2020). However, under conditions of national crisis, the government can set aside the PHAS by enforcing a paragraph of the constitution to install ministerial rule. The government never used this paragraph [52], so two competing discourses found legitimacy in the Swedish national space. One discourse saluted the PHAS: ‘The Swedish openness saves lives in the long run’ (Ludvigsson, DN, 15th April 2020), while a discourse of resistance emerged, arguing for a lockdown in Sweden where the voluntary recommendations were seen as ambiguous and dangerous:

Most Western European countries are already in lockdown. The decision must be made now. It is already late, but it must be done. (Hansson, DN, 15th April 2020).

Again, the image of the nation is discursively repeated when comparisons are made with other countries. In this case, Sweden was compared to other European countries and the Swedish strategy seemed at odds with many countries in the world; some perceived Sweden as being more open and ‘laid-back’. This ‘open’ strategy was questioned by the editor-in-chief of one of the leading papers in Sweden who wrote:

If Sweden lives in the illusion that we are much smarter, then we will have to wait and see. But if we want to be honest, we need to act as quickly as neighbouring countries (Wolodarski, DN, 15th March 2020).

Particular attention and debate related to the closing of the borders of the neighbouring Nordic countries after some weeks of the pandemic, as those borders have long been porous and passport freedom has been applied to Nordic citizens. Within a short period of time, the borders were closed, travel bans were issued and people’s freedom to move was restricted to stop the spread of the virus. Comparisons between the neighbouring Sweden and Denmark were often noted and discussed:

It has hardly escaped anyone that Sweden and the rest of the world have chosen different ways to curb the ravages of the coronavirus. Within the Nordic countries, four countries contrast strict restrictions on public gatherings, mobility, and education against Sweden’s less restrictive stance. In particular, it has become popular to pit Sweden and Denmark against each other. In Sweden, for example, it evoked almost shock-like feelings when Denmark closed its borders to entry on March 14, while the Danish media likes to give the image of an unresponsible Swedish leadership that puts the population’s health at risk by keeping schools, restaurants, and sports facilities open. (Gustafsson, DN, 15th June 2020).

We can see how the discourse on nations, their uniqueness and their ways of reacting were reinforced during the COVID-19 pandemic, with both a ‘banal nationalism’ from above – namely from the state and from PHAS arguing that the virus outbreak should be met by ‘cooperation and joint responsibility, both within and between countries’ – and ‘everyday nationalism’ from some people on the ground arguing for help to ‘flatten the curve’ or to close its borders. Thus, the space of Sweden in relation to the space of the Nordic countries changed through multilayered communication and writings [39] and the Swedish nation’s borders were reinforced. As argued by Rothmüller [51], borders are sites of cultural, social and spatial separation and isolation. During the pandemic, borders were reinforced and spaces outside of the nation could not be reached; this situation heightened fear and an experience of being in a constant state of emergency [51]. However, the Danish writer and social debater Carsten Jensen (DN, 15th April 2020) was given a literary voice in one of the newspapers. Jensen’s text broke in between the two overarching different and contradictory positions of closing or not closing national borders in a strict lockdown. Jensen used a problematizing discourse of individual responsibility, solidarity and caring for others. Here, the ‘we’ was inclusive, formed around ‘the world’ where all are living in a ‘danger zone’:

We have nowhere to escape. We wait in frustration […]. Our self-isolation also means caring for those we don’t know. We don’t isolate ourselves solely to save ourselves but also to save others if we without knowing should become carriers of the disease. (Jensen, DN, 15th April 2020).

However, the discourse of the ‘whole nation’ was a deceptive notion in Sweden, as it was soon recognized that trust in the PHAS authority and the government was significantly lower in some suburban spaces, where many people with non-Swedish backgrounds lived, than it was in the rest of Sweden. Language was identified as a barrier; for example, instructions for ordering a test were primarily in Swedish. Some people did not have a social security number, which was needed to order a COVID test; many lacked smartphones, which were needed to order COVID-19 tests; and many did not have access to a car, which meant they could not attend drive-in testing stations. Thus, a discourse that pertained to socio-spatial implications and suburban space/urban life was articulated that questioned the national strategy, as many inhabitants in several suburban places were transnationally informed. One indication of this was that people in some of the suburban spaces wore masks, while this was very unusual in the city center (Eklund, SvD 15 June 2020). Another articulation of the national discourse was that the spread of the Corona virus was the migrants' fault. However, this argumentation was soon met through a counter-discourse on the ‘double-segregation’ of inhabitants in these areas and questions of a class perspective were raised (Jonsson, DN, 15th November 2020). As several scholars noted, COVID-19 exposed systematic discrimination and social inequities in many places [53,54] and national suburban places and spaces were reconstructed through transnational connections and the discourse of a unified national ‘we’ did not fit this reality [cf. 1, 2]. There are multiple dimensions where place, space and politics are co-constitutive and operate at many levels.

### 6.3 Increasing tensions in the nationalist discourse

As the mortality rate in Sweden increased in March and April 2020, European countries closed their borders towards Sweden. This action again raised questions about failure to handle the COVID-19 virus in Sweden and a discourse close to desperation arose, calling for action from Prime Minister Stefan Löfvén, which was critically commented on in the press:

The mortality rates go up and the Prime Minister Stefan Löfvén needs to show some guts. We started way too late, and we need to catch up. The Swedish model of waiting and seeing what will happen does not work; we cannot continue being reactive. (Hansson, DN, 15th April 2020).

This critical discourse of the Swedish strategy created a vision of how a nation in the north had almost no restrictions during the COVID crisis due to the way Swedish administration was organized. At the same time, some professionals articulated a supportive discourse on the Swedish strategy, claiming that even though mortality rates in Sweden were high, a close-down would be critical for the future:

Calculations show that a total close-down of the society probably would increase the mortality rates in the coming years and also increase the loss of life years in relation to the more open Swedish approach. (Ludvigsson, DN, 15th April 2020).

In May 2020, the overarching and homogeneous discourse of the nation and its loyal citizens was finally torn apart and increasing tensions due to the handling of the pandemic became more clearly articulated and remarked upon:

Many are shocked, while others see a model and sustainable strategy. In focus stands the Swedish form of administration, with roots in the 17th century, which confines the government’s and ministers' power. (Gustafsson, SvD, 15th May 2020).

The Swedish Prime Minister, Stefan Löfvén, was interviewed on national news defending the Swedish corona strategy, which was commented on in the press (Holmström, 15th June 2020). Löfvén claimed that the approach was very similar to that of other countries. Löfven’s message included phrases like ‘it’s not all negative’, ‘some [countries] are interested in how we do it’, and ‘it is too early to draw conclusions’. Löfvén downplayed the risks by highlighting similarities with other countries’ strategies. The prime minister claimed:

It has nothing to do with the strategy. It has to do with other faults in society, faults we now are correcting. You must see if people have died of or with COVID. Here countries report differently. We are very thorough with our reports, so it is too early to draw any definitive conclusions about how the Swedish strategy has worked. (Löfvén, SvD, 15th June 2020).

In the cited formulation, the prime minister argued out from a discourse on Swedish scientific accuracy, stating that ‘we are very thorough with our reports’. This discourse connects to a Swedish science tradition and what Jasanoff [55] introduced as a kind of ‘political culture’, where there is an entanglement of science, national politics, and public knowledge. Interestingly, Hanson et al. [56] showed the stark differences in national health governance during the COVID-19 pandemic in Germany, Sweden, and the UK. Very few public television programmes were provided for scientists to contribute to the national conversation in Sweden, and it was the daily press conferences that gave the PHAS space to report [56 p. 5]. Thus, a national top-down governmental discourse, in the form of a ‘banal’ unified nationalist discourse, was recurring and strong in Sweden. This discursive ‘banal’ nationalist discourse was now and then interwoven with an everyday nationalism from people on the ground [cf. 37].

However, in line with several other countries [56], people in Sweden eventually experienced the handling of COVID-19 as confusing, as the restrictions went from firm to easier almost simultaneously and were largely left up to the individual to follow. In November 2020, the minister of social affairs admitted, ‘As a signal I believe people experienced it [the restrictions] confusing’ as the government first eased the restrictions and then tightened them up again. The confusion was also articulated in the newspapers:

Should society be closed or not? Should measures to reduce the spread be voluntary or mandatory? Society is confronted with a number of extremely hard ethical choices of paths. (Johansson et al., 15th May 2020).

The confusion and insecurity became a struggle that, for people and everyday life in Sweden, included a struggle to handle powerlessness and ‘not knowing’. This discursive restructuring arose from contradictions in social practice, language, and power, which created dilemmas for people in Sweden. Thus, the national space of Sweden became a battle of surviving the unknown threat between the known and the unknown.

## 7 Discussion

### 7.1 Discussion of methodology

The trustworthiness for this study is based on Ricoeur’s thinking that interpreting means choosing not only one but from several possible interpretations [57,58]. Therefore, the interpreter must choose and argue for the most possible interpretation in relation to the aim of the study. As this discourse analysis is qualitative, the results have been presented in a narrative way with descriptive text. This has been considered the best way to capture what is happening in the selected articles. In the following, we use Lincoln and Guba’s four actions: credibility, transferability, dependability, and conformability to discuss trustworthiness [59].

In this study, to increase the trustworthiness related to the methodology, it was firstly a matter of choosing the most appropriate method that could best elucidate the discourse on space and place for health and care in Swedish newspaper articles during the COVID-19 pandemic. Thus, the choice was a CDA approach that provided us with possibilities to capture the role of language in the construction of discourses on COVID-19 [39]. Taking an inductive approach also helped us to first broadly look for patterns in the texts that we could then connect to a framework on space and place drawn from Massey [1, 2], as well as theories on ‘banal’ and ‘everyday’ nationalism [3,4]. Secondly, the fact that the chosen perspective is according to a qualitative tradition means that it is not the amount of data that validate but the transparency of the analysis so that the reader can judge its trustworthiness and the argumentation throughout the analysis. However, data like newspaper articles are not ‘problem-free’, as researchers choose which data to include and which to exclude. The researcher exercises power through the choice and definition of the research topic and the importance of the researcher being self-reflective is stressed [39,60]. It is also important to acknowledge that, when using a qualitative approach, it is not the amount of data that creates credibility. Instead, it is the transparency of the analysis that increases trustworthiness, as overly extensive data material may counteract the clarity of the analysis.

Another way to establish credibility has been to use a suitable method to achieve the aim of the study and to describe the process of the analysis as thoroughly as possible. The pre-understanding has been recognized by letting the research team take part in, discuss, and question the analysis and results throughout the analytical process.

Transferability in this kind of qualitative study can be hard to accomplish, as the result must be viewed and understood within the context and geographical area. Therefore, it has been important to describe the inclusion criteria, data collection methods, and time period over which the data were collected. Thus, readers can determine how confident they can be about transferring the results to other situations.

Dependability is closely tied to credibility [59]. In qualitative research, it is usually not possible to calculate dependability quantitatively. As the present study has a qualitative focus, the dependability has instead focused on presenting how we have gone about collecting and processing data in an interesting, reliable and comprehensible way.

Finally, we attempted to ensure that confirmability included being open-minded and trying to set aside possible pre-understanding notions about the data material, as well as using quotations from the articles as a way to visualize what was happening in the chosen articles.

### 7.2 Discussion of findings

Given the unique nature of COVID-19 – a pandemic that affected most of the world – the aim of the present study was to explore in-depth how discourses on place and space for health and care was discursively constructed in the two main Swedish national newspapers during the early stages of COVID-19 pandemic in Sweden in 2020. Not unexpectedly, this event is generating an ever-increasing amount of research in various disciplines. We add to this research, particularly to the fields of nationalism and health showing how discourses on space, place, health and care in Swedish newspapers constructed the pandemic during the initial year 2020 as a global threat leading to a reinforcement of ‘us and them’ and various boundary settings. However, the more time passed, increasing tensions in the nationalist discourse was revealed, and has likely challenged the Swedish self-image and the degree of Swedish exceptionalism in areas such as welfare state politics, health and nursing, and political behaviour. Swedish exceptionalism and its decline were already discussed by Pierre in 2015 [11]. Such discussions might now increase.

In this study, we identified three broad discourses and how they relate to space, place, health and care: (1) united against the threat – loyalty in the national space, (2) reconstructing national borders and the Swedish strategy debate, and (3) increasing tensions in the nationalist discourse.

First, at the onset of the COVID-19 outbreak, the pandemic was identified as a global threat that did not take account of any borders, and Swedish society was put under severe stress. Starting in March 2020, this ‘unknown threat’ led to a constant state of national emergency up to February 2023 [cf. 51, 60], but with slightly different intensity at certain periods. Together with earlier research [7,61,62], we see how this ‘state of emergency’, initially in March–April 2020, formed a strong unifying nationalist discourse in the analysed newspapers. Following Fairclough, this ‘order of discourse’ built on images of the Swedish nation as ‘our home’, demanding citizens to demonstrate loyalty to the recommendations brought forward by PHAS. The health and care during the pandemic were described as a ‘struggle for the whole of Sweden’ and people were told that Sweden needed to unite in this ‘struggle’.

Second, the repeated discursive construction in the press of an inhabitant in Sweden was a ‘loyal’ and ‘responsible person’ included in a ‘responsible nation’. Thus, the nation turned into a symbiosis of the Swedish inhabitant in an overarching and dominating ‘banal nationalist’ discourse. The nation was viewed as self-evident, as a natural and bounded place through the language of ‘we’, ‘us’, ‘our’ and ‘here’. The nation was internally remade through intensification of state administration and experts, which helped to produce the idea of a bounded and unified nation, as earlier shown in work on nationalism [3,5,61]. Thus, there was a strong ‘banal nationalist’ order of discourse, located in scientific expertise and connected to the Swedish kind of ‘political culture’ where science, national politics and public knowledges are entangled [55, cf. 39]. This political culture led Sweden to choose a strategy that was seen as being different from nearly all other European countries, which attracted both praise and criticism [7]. The PHAS press conferences and the lengthy discussions and tensions of the Swedish strategy, both reproduced and challenged the self-image of Sweden as ‘the most modern country in the world’ [11,21].

Third, during the first year of COVID-19 it was also clear that people in Sweden eventually found the handling of COVID-19, as PHAS recommended, confusing. People were pushed back and forth from firm to easier restrictions and it was up to themselves how they managed the restrictions. This occurred not only in Sweden; Balog-Way and McComas wrote that the pace at which leading risk communicators in different countries changed their messages during COVID-19 was on a high level [63]. Thus, people’s uncertainty in handling powerlessness and ‘not knowing’, along with mixed messages, might result in a lack of trust, something that future research needs to work on. As trust can be seen as a main determinant of peoples' compliance and collaboration with governmental measures during a pandemic [63], unifying nationalism was at stake. Moreover, the recommendations for everyday behaviour and living were in line with the view that such recommendations were possible to communicate and comply with throughout the whole of Sweden, which was later shown not to be the case. There were ‘pockets of places’ where the virus spread dramatically, mostly due to the fact that people living in many suburban areas could not stay home from work; this had devastating results for the diffusion of the virus, and for health and care in these areas [25]. Thus, we see how places differ within the same space and how places are the product of social relations rather than fixed and stable, as argued by Massey [1]. On the other hand, Sweden as a space became dramatically closed through the power of the nationalist discursive events, with particular articulations related to different forms of borders. The Swedish national border and the national space were constructed in relation to neighbouring nations, but borders were also constructed to specific urban places and spaces within Sweden, as well as personal borders in relation to family life. Sweden as a nation, along with its inhabitants and its healthcare organisation, were reconstructed in a way that moved the figure of homeliness to the background and switched estrangement to be the figure. The figure of estrangement points toward a discourse of divagation between what was taken for granted concerning health and care before the virus. Health was no longer an option, nor was the home as secure.

In summary, this study offers insights into the discourse of nations, their uniqueness and how their ways of reacting were reinforced during the COVID-19 pandemic, through both ‘banal nationalism’ and ‘everyday nationalism’. The space of Sweden in relation to the space of the Nordic countries was greatly challenged and changed, and the Swedish nation’s space and borders were reinforced according to an earlier nationalist model. The analysis shows how the pandemic clearly demonstrated that spaces and places had the capacity to not only ensure health but also to threaten it. Moreover, we see how this overarching ‘order of discourse’ is challenged in actual discursive events and how discursive reconstructions took place, which led to contradictions and confusion and generated dilemmas for people. The legitimacy and power of the modern state and its institutions were challenged in discourse, and we see how dialectical relations among the state, media and the Swedish people bring meaning and make meaning to the complex relations that constitute social life.

Our results indicate the importance of policymakers and politicians ensuring sustainable reforms and organisation in public health work – especially consequences for health, but also on health communication due to space and place – both on a national level and in relation to an international arena. As argued by the former Swedish state epidemiologist at PHAS, Anders Tegnell (interview 2023-11-09) [64], after the COVID-19 pandemic ‘there will be new pandemics, but it is impossible to say when’.

## 8 Conclusion

This study has sought to shed light on discourses of the Swedish nation, its uniqueness and how ways of reacting were reinforced during the COVID-19 pandemic, through both ‘banal nationalism’ and ‘everyday nationalism’. Thus, the space of Sweden in relation to the space of the Nordic countries was greatly challenged, and the Swedish nation’s space and borders were reinforced according to an earlier nationalist model. The analysis shows how the pandemic clearly demonstrated that spaces and places had the capacity to not only ensure health but also to threaten it. Moreover, we see how the overarching ‘order of discourse’, ‘United against the threat – loyalty in the national space’ is challenged in actual discursive events and that a discursive reconstruction took place. This led to contradictions and confusion and generated dilemmas for people. The legitimacy and power of the Swedish state and its institutions were challenged in discourse, which led to the questioning of the Swedish self-image and ‘the Swedish model’ as a modern, unique, well-functioning and superior state [21]. Future research could focus specifically on what role nationalist discourses played for discursive framings of the Swedish strategy, the role of media during COVOD-19 and, on a more general level, the role of nationalism related to space, place, health, and care, and possibly a new phase of development of nationalism as argued by Triandafyllidou [34].

The authors confirm that the data supporting the findings of this study are available within the article [and/or] its supplementary materials.

## CRediT authorship contribution statement

**K. von Brömssen:** Writing – review & editing, Writing – original draft, Methodology, Formal analysis, Conceptualization. **Å. Roxberg:** Writing – review & editing, Writing – original draft, Methodology, Formal analysis, Conceptualization. **C. Werkander Harstäde:** Writing – review & editing, Writing – original draft, Methodology, Formal analysis, Data curation, Conceptualization.

## Declaration of competing interest

The authors declare that they have no known competing financial interests or personal relationships that could have appeared to influence the work reported in this paper.

## Footnotes

1 The research network “Space and Place for Health” was established in 2017 as an interdisciplinary research network that explore relations between space, place, health, and care, both methodologically and theoretically. The network has so far published a discussion article (2020) in the field titled “Space and place for health and care” The aim of the network is to contribute to a greater understanding of the state of the art of research engaged with conceptual matters of space and place for health and care”. Professor Åsa Roxberg, is the leader of the network.
